# Ferric Carboxymaltose as Treatment in Women with Iron-Deficiency Anemia

**DOI:** 10.1155/2017/9642027

**Published:** 2017-04-13

**Authors:** Melvin H. Seid, Angelia D. Butcher, Ashwin Chatwani

**Affiliations:** ^1^Unified Women's Clinical Research, 1100-C South Stratford Road, Suite 310, Winston-Salem, NC 27103, USA; ^2^Luitpold Pharmaceuticals, Inc., 800 Adams Ave, Suite 100, Norrisville, PA 19403, USA; ^3^Department of Obstetrics and Gynecology, Temple University School of Medicine, Philadelphia, PA 19140, USA

## Abstract

*Objective*. To evaluate safety and efficacy of intravenous ferric carboxymaltose (FCM) versus standard medical care (SMC) for iron-deficiency anemia (IDA) in postpartum women and women with heavy menstrual bleeding.* Study Design*. This open-label, multicenter study randomized women with IDA (hemoglobin ≤ 11.0 g/dL) to single doses of FCM (15 mg/kg [maximum 1000 mg]) or SMC (this treatment was determined by the investigator and there may have been no treatment). Safety data (primary outcome) were collected for 30 days.* Results*. Of 2045 subjects enrolled (FCM: *n* = 1023; SMC: *n* = 1022), 996 received FCM and 1022 received SMC. At least 1 serious adverse event (AE) was reported by 0.6% and 2.2% of subjects in the FCM and SMC groups, respectively; none were considered treatment related. The difference in serious AEs was primarily due to higher rates of uterine leiomyoma, uterine hemorrhage, and menorrhagia in SMC subjects with heavy menstrual bleeding. Common AEs were generally predictable, with higher rates of infusion site reactions in FCM subjects and gastrointestinal AEs in SMC subjects. Mean hemoglobin increases were greater in the FCM group than the SMC group.* Conclusion*. FCM was well tolerated and effectively increased mean hemoglobin levels in postpartum women or women with heavy menstrual bleeding and IDA. This trial is registered with ClinicalTrials.gov, NCT00548860.

## 1. Introduction

Iron deficiency is the most common cause of anemia [1, 2]. Women who are of reproductive age are particularly at risk owing to blood loss or increased iron demand attributed to menstruation, pregnancy, and lactation [3, 4]. Iron-deficiency anemia (IDA) is associated with adverse effects on cognitive function, physical activity, immune response, and pregnancy outcome. The World Health Organization defines IDA as hemoglobin < 12.0 g/dL in women who are not pregnant and <11.0 g/dL in women who are pregnant [4, 5]. Postpartum anemia reportedly affects up to 27% of women, and approximately 20% of women suffer excessive menstrual blood loss [3, 6]. IDA associated with childbirth or heavy menstrual bleeding can be potentially reversed with iron repletion therapy [7]. Iron therapy for IDA has been shown to reduce morbidity by improving physical activity and reducing fatigue and cognitive deficits [3, 4, 7].

Iron therapy can be administered orally or parenterally [3, 4, 8]. Oral iron is the treatment of choice for the majority of patients with IDA because it is safe, effective, inexpensive, and readily available. However, the tolerability of oral iron therapy can be problematic, with up to 40% of patients reporting gastrointestinal adverse events (AEs) [3, 9]. In addition, sustained response to oral iron treatment may be complicated by ongoing losses from heavy menstrual bleeding that exceed the gastrointestinal absorption of iron [8]. Parenteral iron administration is preferred for patients who cannot tolerate or are unresponsive to oral iron treatment and for patients who are unable to absorb sufficient iron in the gastrointestinal tract or for whom blood transfusions should be avoided [4, 8]. Intravenous (IV) low-molecular-weight iron dextran has been associated with an incidence of anaphylaxis or anaphylactoid reactions as high as 1.7% [10, 11]. The high incidence of these serious AEs is believed to be caused by the formation of antibodies to the dextran moiety. Newer parenteral iron products (iron sucrose and iron gluconate) do not contain the dextran moiety, and the incidence of anaphylaxis with these products is markedly lower [11, 12]. However, the physical characteristics of iron gluconate and iron sucrose limit the dose and administration rate.

Ferric carboxymaltose (FCM) is a Type I polynuclear iron (III)-hydroxide carbohydrate complex that produces a slow and controlled delivery of the complexed iron to endogenous iron binding sites [13, 14]. In randomized trials in postpartum women with anemia and women with heavy menstrual bleeding, treatment with FCM significantly improved hemoglobin levels compared with oral iron treatment [15–17]. In previous trials in women with IDA in the postpartum period or women with heavy menstrual bleeding, the total dose of FCM was individualized to restore a calculated iron deficit at baseline, with total FCM doses up to 2500 mg administered weekly over several weeks. Since the total iron requirement was found to be ≤1600 mg for approximately 90% of postpartum women or women with heavy menstrual bleeding in these studies, the data suggest that 1 to 2 doses of FCM 15 mg/kg would likely treat most of these subjects. Therefore, the objective of the current large, randomized clinical trial was to evaluate the safety and efficacy of a more convenient, single 15 mg/kg dose (maximum 1000 mg) of FCM compared with standard medical care (SMC) for treatment of IDA in women with IDA in the postpartum period or women with heavy menstrual bleeding.

## 2. Material and Methods

### 2.1. Study Design

An open-label, randomized study was conducted at 130 sites and consisted of a screening visit and 2 study visits at day 0 and day 30. Each site received institutional review board approval before initiation of the study, and informed consent was obtained from each subject prior to participation. This study was conducted in compliance with Good Clinical Practice guidelines and the Declaration of Helsinki.

### 2.2. Subjects

Women with IDA in the postpartum period were eligible to participate if they had hemoglobin levels ≤  11.0 g/dL obtained at least 18 hours after delivery. Women with heavy menstrual bleeding were eligible if they had hemoglobin levels ≤ 11.0 g/dL or point-of-care hemoglobin levels ≤ 11.5 g/dL. Heavy menstrual bleeding was defined as ≥1 of the following within the preceding 6 months: (a) inability to control flow with tampons alone, (b) use of >12 pads per period or 4 tampons per day unless subject was unusually fastidious, (c) passage of clots, or (d) period duration >7 days.

Exclusion criteria included recent (≤3 months) gastrointestinal bleeding or significant acute blood loss (other than at delivery); a history of anemia other than IDA due to pregnancy/delivery or heavy menstrual bleeding; current treatment with myelosuppressive therapy or asthma therapy; and recent administration (≤1 month) of blood transfusion, IV iron, erythropoietin, or investigational drug. Women who were pregnant or had a history of hypersensitivity to FCM or were planning surgery or had an active infection including hepatitis B or C, known human immunodeficiency virus seropositivity, a history of malignancy, hemochromatosis, significant cardiovascular disease, elevated liver enzymes, alcohol abuse, or drug abuse were also excluded from study participation. Subjects were also excluded from participation on day 0 if they had point-of-care hemoglobin levels > 12.0 g/dL or a systolic blood pressure > 160 or <80 mm Hg or a diastolic blood pressure > 100 or <40 mm Hg prior to randomization.

### 2.3. Treatment

Subjects were randomized in a 1 : 1 ratio using a central interactive voice response system to receive either IV FCM or SMC (treatment in the SMC group was determined by the investigator for individual patients and there may have been no treatment). Randomization was stratified by etiology of IDA (postpartum or heavy menstrual bleeding); baseline hemoglobin levels ≤ 8.0, 8.1 to 9.5, or >9.5 g/dL; cardiac risk category (low or high); and past response to oral iron (poor or not poor). Subjects with 1 or no cardiac risk factors were designated as having low cardiac risk. Subjects with 2 or more cardiac risk factors, including smoking, high blood pressure, high blood cholesterol, diabetes, overweight or obesity, physical inactivity, and family history of heart disease (father/brother before age 55 years or mother/sister before age 65 years), were designated as having high cardiac risk.

Subjects in the FCM group received FCM 15 mg/kg (up to a maximum of 1000 mg) in a slow IV infusion over 15 minutes on day 0. Prepregnancy weight was used to determine the FCM dose for postpartum subjects. Sitting pulse and blood pressure were monitored before, immediately after, and at 30 and 60 minutes after administration of IV iron.

Subjects in the SMC group were treated for IDA as determined by the investigator from day 0 through day 30. Ferrous sulfate 325 mg tablets (65 mg of elemental iron) were provided to the subject as study medication if the investigator determined it to be the best method of treatment. All subjects randomized to receive SMC were considered to have started treatment, as no treatment was an option.

### 2.4. Study Assessments

Safety was assessed by measurement of vital signs and findings on physical examination in addition to hematology, chemistry, and iron indices determined from blood samples obtained at day 0 and day 30. Treatment-emergent AEs were recorded from administration of study drug for FCM-treated subjects and after randomization for SMC subjects through completion of the study (day 30) or 30 days after last dose of study drug, whichever was later. When subjects returned to the clinic on day 30, AEs were elicited by use of nonspecific questions. Subjects were encouraged to report AEs at their onset, and all AEs, whether elicited, reported spontaneously, or observed by the physician or study staff, were recorded with date of onset, relationship to study medication based on investigator opinion, action taken, and date of resolution if the AE resolved. Allergic reactions were classified according to the National Cancer Institute Common Terminology Criteria for Adverse Events version 3.0, including grading of all events to quantify severity. Follow-up telephone calls to collect AEs were made 30 days after the last dose of study drug to all FCM-treated subjects who terminated early and 30 days after last dose of study drug to all SMC subjects.

### 2.5. Study Endpoints and Analyses

The primary goal of this study was to assess safety. The safety population included all FCM-treated subjects who received a dose of FCM on day 0 and all randomized SMC subjects. A sample size of 750 subjects per group provided approximately 80% power to detect a statistically significant difference in the incidence of serious AEs, when the incidence was 5.25% for FCM and 2.5% for SMC. The primary endpoint was the incidence of serious AEs, including death, hospitalization, disability, congenital anomaly/birth defect, and life-threatening events. Other safety endpoints included incidence and severity of AEs and treatment-emergent abnormal clinical laboratory values. Quantitative endpoints were summarized descriptively using mean, standard deviation, median, and range, and investigators judged the clinical significance of laboratory abnormalities. Differences between treatment groups were assessed using Fisher's exact test. All tests were 2-tailed with 0.05 alpha level. Efficacy was assessed as the mean change from baseline to highest postrandomization value for hemoglobin and ferritin levels, which were compared by cause of anemia across treatment groups and use of 1-way analysis of variance.

## 3. Results

### 3.1. Study Participants

**Figure 1:**
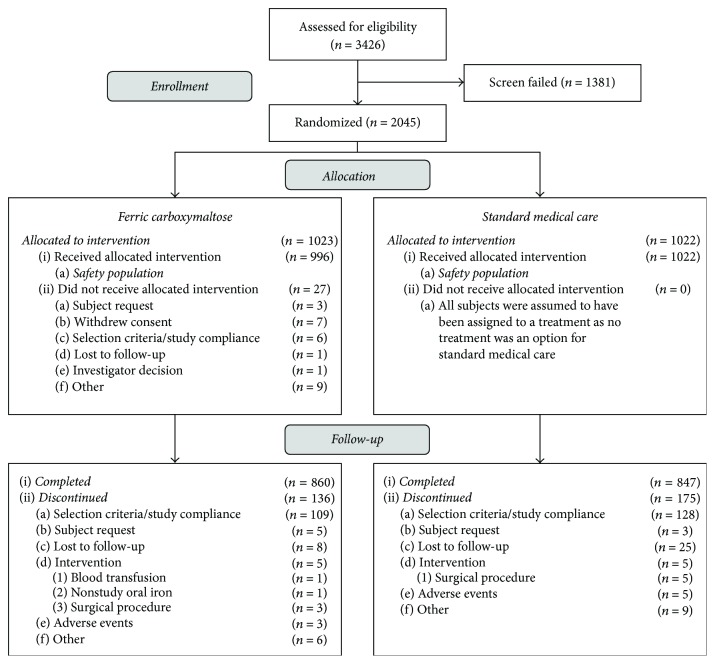


A total of 2045 women were randomized to receive FCM (*n* = 1023) or SMC (*n* = 1022) (Figure 1). In the FCM group, 27 subjects discontinued the study before dosing and were excluded from the analysis. Reasons for discontinuation are summarized in Figure 1. No subjects randomized to receive SMC were excluded from the analysis. Thus, the safety population included 996 subjects in the FCM group and 1022 subjects in the SMC group. More than 80% of subjects in both treatment groups completed the study through day 30, including 860 (84.1%) in the FCM group and 847 (82.9%) in the SMC group. Reasons for discontinuation were similar between treatment groups (Figure 1). No notable differences were observed between the treatment groups for any demographic and baseline characteristic (Table 1). The cause of IDA was blood loss during delivery and/or antepartum IDA in approximately 60% of subjects and heavy menstrual bleeding in 40% of subjects. The majority of subjects did not receive prior iron treatment (FCM: 74.7%; SMC: 72.3%).

The mean dose of FCM during the study was 944 mg for all subjects, 926 mg for postpartum subjects, and 970 mg for subjects with heavy menstrual bleeding. Two subjects received an incorrect dose of FCM owing to the use of an incorrect body weight for weight-based dosing; in both cases, the incorrect dose was ≤1000 mg of FCM. Oral iron therapy (typically ferrous sulfate) was initially prescribed for 93% of subjects randomized to SMC; no treatment was received by 2.4% of SMC subjects. The SMC subjects who received oral ferrous sulfate as study medication had a mean compliance rate of 95.2%. Based on the number of pills taken versus the number of pills prescribed, similar prescribing and compliance rates in the SMC subjects were observed between the postpartum and heavy menstrual bleeding populations.

### 3.2. Safety

The incidence of serious AEs was statistically significantly higher among subjects in the SMC group (22/1022 [2.2%]) than among those in the FCM group (6/996 [0.6%]; *P* = 0.004). The investigators did not consider any serious AE in either treatment group to be related to study medication. In the FCM group, the incidence of serious AEs was similar for postpartum subjects and subjects with heavy menstrual bleeding (0.7% and 0.5%, resp.; *P* > 0.9999). In the SMC group, subjects with heavy menstrual bleeding had a higher incidence of serious AEs than postpartum subjects (4.3% and 0.8%, resp.; *P* = 0.0003). The increased incidence of serious AEs in the subjects with heavy menstrual bleeding receiving SMC was primarily due to higher rates of uterine leiomyoma, uterine hemorrhage, and menorrhagia.

Seven (0.7%) subjects in the FCM group and 22 (2.2%) subjects in the SMC group discontinued study drug early owing to the occurrence of AEs (Table 2). The proportion of subjects who discontinued study drug owing to AEs was similar between those with postpartum anemia and those with heavy menstrual bleeding in each treatment group.

At least 1 AE was experienced by 27.3% (272/996) of subjects in the FCM group and 26.9% (275/1022) of subjects in the SMC group (*P* = 0.841). The incidence of injection site reactions (i.e., extravasation, pain, bruising, irritation, paresthesia, and coldness) was higher in the FCM group, and the incidence of gastrointestinal AEs (i.e., constipation, diarrhea, nausea, and vomiting) was higher in the SMC group (Table 3). Subjects in the FCM group had a significantly higher incidence of hypophosphatemia, dysgeusia, increased alanine aminotransferase, and immune system disorders (hypersensitivity, *n* = 4; latex allergy, *n* = 1) than subjects in the SMC group.

AEs are summarized separately in Table 4 for postpartum subjects and those with heavy menstrual bleeding within each treatment group. A larger percentage of subjects with heavy menstrual bleeding compared with postpartum subjects reported ≥1 AE in the FCM group (33.1 and 23.6%, resp.; *P* = 0.0013) and in the SMC group (31.3% and 24.1%, resp.; *P* = 0.0115). In both treatment groups, postpartum subjects and those with heavy menstrual bleeding had statistically significantly different rates of gastrointestinal events (FCM: *P* = 0.0072; SMC: *P* = 0.0013) and general disorders and administration site conditions (FCM: *P* = 0.0285; SMC: *P* = 0.0002).

Four subjects (0.4%) in the FCM group (2 postpartum and 2 heavy menstrual bleeding) and no subjects in the SMC group experienced hypersensitivity reactions during the study. All hypersensitivity events were Grade 1 in severity. Two subjects in the FCM group (1 postpartum and 1 heavy menstrual bleeding) were reported to have hypotension during the study; both AEs were Grade 1 in severity, and neither subject had a history of hypotension.

A significantly greater proportion of subjects in the FCM group than in the SMC group had a transient decrease in serum phosphorus (9.0% versus 0%; *P* < 0.001). The difference was more pronounced in subjects with heavy menstrual bleeding; 21.3% of subjects with heavy menstrual bleeding in the FCM group had a transient decrease in serum phosphorus compared with only 0.7% of subjects with postpartum anemia. These changes were not associated with clinical symptoms. No other treatment-emergent abnormal clinical chemistry values were observed.

### 3.3. Efficacy

**Figure 2:**
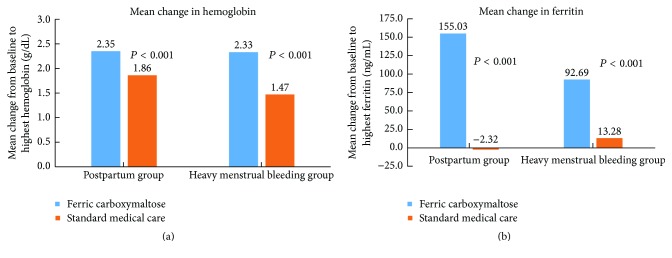


Subjects treated with FCM and SMC experienced improvements in hemoglobin from baseline to the highest value during treatment, and subjects treated with FCM with either postpartum anemia or heavy menstrual bleeding experienced significantly greater mean increases in hemoglobin and ferritin levels from baseline to highest value compared with subjects treated with SMC (Figure 2 and Table 5). Mean hemoglobin levels at baseline were higher in postpartum subjects than in subjects with heavy menstrual bleeding (FCM: 10.2 versus 9.3 g/dL, *P* < 0.001; SMC: 10.1 versus 9.4 g/dL, *P* < 0.001). In the FCM group, the mean increase in hemoglobin from baseline to highest value was similar between subjects with postpartum anemia and those with heavy menstrual bleeding (2.35 versus 2.33 g/dL; *P* = 0.808). In the SMC group, the mean increase in hemoglobin was greater in postpartum subjects than in subjects with heavy menstrual bleeding (1.86 versus 1.47 g/dL; *P* < 0.001).

In both treatment groups, mean ferritin levels at baseline were higher in postpartum subjects than in subjects with heavy menstrual bleeding (FCM: 25.99 versus 8.89 ng/mL, *P* < 0.001; SMC: 26.55 versus 7.95 ng/mL, *P* < 0.001). In the FCM group, the mean increase in ferritin from baseline to highest value was larger in subjects with postpartum anemia than in subjects with heavy menstrual bleeding (155.03 versus 92.69 ng/mL; *P* < 0.001). In the SMC group, a mean decrease was observed in the subjects with postpartum anemia compared with a mean increase observed in subjects with heavy menstrual bleeding (−2.32 versus 13.28 ng/mL; *P* < 0.001).

## 4. Discussion

Similar to 4 previous randomized, controlled trials of FCM [15–18], this large, open-label, randomized study supports that a single 15 mg/kg dose of IV FCM was safe and well tolerated for the treatment of subjects with IDA in the postpartum period and subjects with IDA caused by heavy menstrual bleeding. The current study evaluated the safety and efficacy of a more convenient, single 15 mg/kg dose (maximum 1000 mg) of FCM. Overall, the incidence of serious AEs was low in the present study. Differences in rates of common AEs between treatment groups were generally predictable, with a higher incidence of infusion site reactions (i.e., extravasation, pain, bruising, irritation, paresthesia, and coldness) in the FCM group and a higher incidence of gastrointestinal AEs (i.e., constipation, diarrhea, nausea, and vomiting) in the SMC group. Subjects in the SMC group experienced more serious AEs than subjects in the FCM group; however, none of the reported serious AEs were considered related to study medication. Subjects with heavy menstrual bleeding who received SMC had an increased incidence of AEs primarily owing to higher rates of uterine leiomyoma, uterine hemorrhage, and menorrhagia in this subject subset. Comparison between the safety and efficacy of subjects with postpartum IDA and those with heavy menstrual bleeding was possible due to the fact that treatment randomization was stratified by etiology of anemia. Among postpartum women, the most common AEs were increased transaminase levels and injection site extravasation in the FCM group and constipation and nausea in the SMC group. Women with heavy menstrual bleeding experienced similar common adverse events, with headache, injection site extravasation, injection site pain, and dizziness in the FCM group and constipation, nausea, diarrhea, and vomiting in the SMC group.

Four subjects in the FCM group experienced Grade 1 hypersensitivity reactions, and no subject experienced anaphylaxis or an anaphylactoid reaction. Based on previous studies conducted on low-molecular-weight iron dextran the rate of anaphylaxis or anaphylactoid reactions was reported to be 1.7% [10, 11]. While the present study is not an iron dextran investigation, if we use this rate of anaphylaxis or anaphylactoid reactions as a guide, the probability of observing ≥1 reaction among 1023 subjects was >99.9%.

Barish and colleagues published findings of a single dose (*n* = 735) and multidose (*n* = 703) safety trial of FCM versus SMC (including approved oral or IV iron preparations) for the treatment of IDA associated with a variety of medical conditions including gastrointestinal disorders, chronic kidney disease, heavy menstrual bleeding, and postpartum-related anemia [19]. The maximum single dose of FCM was 750 mg compared with 1000 mg used in the current study. FCM was well tolerated, and there were no clinically important differences in safety outcomes between the 2 groups. Supporting the results of the current study, the incidence of AEs was similar between the FCM and SMC treatment groups in the Barish et al. study, with more gastrointestinal AEs reported in the SMC group.

We observed a higher incidence of hypophosphatemia in the FCM group compared with the SMC group. The majority of hypophosphatemia events occurred in women with heavy menstrual bleeding who received FCM. Transient decreases in serum phosphorus have been reported in other studies of FCM [15–17, 20]. In a study evaluating the efficacy of FCM for women with heavy menstrual bleeding, 70% of subjects receiving FCM experienced a transient decrease in serum phosphorus [16]. The authors hypothesized that the transient decrease is due to an observed increase in the full length (uncleaved) form of fibroblast growth factor 23, an osteocyte-derived hormone that regulates phosphate and vitamin D homeostasis [20, 21]. The effects of FCM on serum phosphorus are transient and appear to be unassociated with other clinically significant events.

Similar to the previously reported trials, efficacy outcomes in the current trial demonstrated that FCM was superior to SMC in improving hemoglobin and correcting IDA [15–17]. Treatment differences were smaller than those reported for previously published trials in postpartum subjects and subjects with heavy menstrual bleeding [16, 17]. This is likely explained by the higher baseline hemoglobin values for postpartum subjects in this trial, as specified by the inclusion criteria. Furthermore, the total dose of FCM was generally higher in the prior studies, as the FCM dose was individualized by using the Ganzoni method to calculate the total amount of iron needed for repletion, which was typically administered in up to 3 infusions [15–17]. In this study, all subjects randomized to receive FCM were given a single dose of 15 mg/kg, not to exceed 1000 mg. FCM was also superior in replenishing tissue stores of iron as measured by ferritin levels compared with SMC. The results of this trial are consistent with others demonstrating that oral iron does not restore tissue iron stores in this population [15–18]. The mean decrease in ferritin for women with heavy menstrual bleeding in the SMC group is consistent with an inability of oral iron to replenish tissue stores of iron. The finding suggests that oral intake of iron may be insufficient for many women with heavy menstrual bleeding to replace the rapid loss of iron from menstruation.

The safety and efficacy of FCM compared with currently available IV iron could not be assessed in this trial because more than 90% of subjects in the SMC group received oral iron. In a noninferiority study in 2584 subjects, FCM was found to be noninferior to IV iron sucrose in subjects with non-dialysis-dependent chronic kidney disease [22]. In another study of subjects with a broad range of IDA etiologies, 503 subjects were randomly assigned to receive FCM versus standard of care IV iron [22]. FCM was superior to the IV iron standard of care in increasing hemoglobin levels. FCM was given as 2 injections of 750 mg separated by 1 week in both of these trials.

In conclusion, this study demonstrated that a single dose of FCM up to 1000 mg, given by IV infusion, is safe and effective in the treatment of IDA in postpartum women and women with heavy menstrual bleeding. FCM improves hemoglobin levels and replenishes iron stores more effectively than oral iron.

## Figures and Tables

**Table 1:** Demographic and baseline characteristics (safety population).

	FCM (*n* = 996)	SMC (*n* = 1022)
*Demographic characteristics*		
Mean (SD) age, years	31.2 (9.36)	31.4 (8.98)
Race, *n* (%)		
White	457 (45.9)	477 (46.7)
African American	391 (39.3)	387 (37.9)
Hispanic	122 (12.2)	135 (13.2)
Asian	5 (0.5)	8 (0.8)
Other	21 (2.1)	15 (1.5)
Mean (SD) height, cm^a^	163.4 (6.93)	163.1 (7.07)
Mean (SD) weight, kg^b^	82.0 (21.71)	82.8 (21.64)
Mean (SD) prepregnancy weight, kg^c^	72.4 (19.96)	73.3 (19.85)
*Baseline characteristics*		
Etiology of anemia, *n* (%)		
Postpartum	606 (60.8)	623 (61.0)
Heavy menstrual bleeding	390 (39.2)	399 (39.0)
Cardiac risk factor, *n* (%)^d^		
Low	709 (71.2)	723 (70.7)
High	287 (28.8)	299 (29.3)
Poor response to oral iron, *n* (%)^e^		
Yes	236 (43.1)	252 (44.4)
No	312 (56.9)	316 (55.6)
Mean (SD) hemoglobin, g/dL^f^	9.9 (1.32)	9.8 (1.29)

FCM = ferric carboxymaltose; SD = standard deviation; SMC = standard medical care.

^a^ *n* = 996 for ferric carboxymaltose and *n* = 1019 for standard medical care.

^b^ *n* = 992 for ferric carboxymaltose and *n* = 1013 for standard medical care.

^c^Prepregnancy weight for postpartum subjects only; *n* = 609 for ferric carboxymaltose and *n* = 625 for standard medical care.

^d^If a subject had ≤1 cardiac risk factor (specifically, smoking, high blood pressure, high blood cholesterol, diabetes, overweight or obese, physical inactivity, or family history of heart disease), the subject was categorized as low cardiac risk; if the subject had ≥2 risk factors, they were categorized as high cardiac risk.

^e^ *n* = 548 for ferric carboxymaltose and *n* = 568 for standard medical care.

^f^ *n* = 994 for ferric carboxymaltose and *n* = 1019 for standard medical care.

**Table 2:** Adverse events leading to premature discontinuation of study drug by treatment group.

FCM (*n* = 996)		SMC (*n* = 1022)	
*System organ class*		*System organ class*	
Adverse event	*n* ^a^ (%)	Adverse event	*n* ^b^ (%)

*General disorders and administration site conditions*		*Gastrointestinal disorders*	
Injection site extravasation	5 (0.5)	Constipation	6 (0.6)
Injection site bruising	2 (0.2)	Nausea	6 (0.6)
*Immune system disorders*		Abdominal pain	5 (0.5)
Hypersensitivity	1 (0.1)	Vomiting	2 (0.2)
		Abdominal discomfort	1 (0.1)
		Small intestinal obstruction	1 (0.1)
		Abdominal hernia	1 (0.1)
		Abdominal adhesions	1 (0.1)
		*Reproductive system and breast disorders*	
		Uterine hemorrhage	2 (0.2)
		Menorrhagia	1 (0.1)
		Postpartum hemorrhage	1 (0.1)
		Vulvovaginal pruritus	1 (0.1)
		Drug exposure via breast milk	1 (0.1)

FCM = ferric carboxymaltose; SMC = standard medical care.

^a^One subject had multiple adverse events leading to premature discontinuation of study drug.

^b^Six subjects had multiple adverse events leading to premature discontinuation of study drug.

**Table 3:** Treatment-emergent adverse events occurring in ≥2% of subjects either in treatment group or with a statistically significant^a^ difference between the FCM and SCM treatment groups (safety population).

System organ class^b^ Preferred term	FCM (*n* = 996) *n* (%)	SMC (*n* = 1022) *n* (%)
*≥1 treatment-emergent AE*	*272 (27.3%)*	*275 (26.9)* ^c^
*Gastrointestinal disorders*	34 (3.4)	137 (13.4)
Constipation	9 (0.9)	79 (7.7)
Diarrhea	9 (0.9)	20 (2.0)^c^
Nausea	8 (0.8)	35 (3.4)
Vomiting	2 (0.2)	13 (1.3)
*General disorders and administration site conditions*	87 (8.7)	12 (1.2)
Injection site extravasation	24 (2.4)	0
Injection site pain	12 (1.2)	1 (0.1)
Injection site bruising	11 (1.1)	0
Injection site irritation	8 (0.8)	0
Injection site paresthesia	6 (0.6)	0
Injection site coldness	5 (0.5)	0
*Immune system disorders*	5 (0.5)	0
*Investigations*	25 (2.5)	11 (1.1)
ALT increased	18 (1.8)	6 (0.6)
*Metabolism and nutrition disorders*	8 (0.8)	1 (0.1)
Hypophosphatemia	6 (0.6)	0
*Nervous system disorders*	49 (4.9)	21 (2.1)
Headache	25 (2.5)	15 (1.5)^c^
Dysgeusia	7 (0.7)	0

AE = adverse event; ALT = alanine aminotransferase; FCM = ferric carboxymaltose; SMC = standard medical care.

^a^All comparisons between the FCM and SMC groups are statistically significant (*P* ≤ 0.05) unless otherwise noted.

^b^Each subject is counted only once per system organ class.

^c^Not statistically significant from the FCM group.

**Table 4:** Treatment-emergent adverse events occurring in ≥2% of subjects in either treatment group or with a statistically significant difference^a^ between the FCM or SMC treatment groups by anemia etiology (safety population).

System organ class^b^ Preferred term	FCM		SMC	
	Postpartum (*n* = 606) *n* (%)	HMB (*n* = 390) *n* (%)	Postpartum (*n* = 623) *n* (%)	HMB (*n* = 399) *n* (%)
*≥1 treatment-emergent AE*	*143 (23.6)*	*129 (33.1)*	*150 (24.1)* ^c^	*125 (31.3)* ^c^
*Gastrointestinal disorders*	13 (2.1)	21 (5.4)	66 (10.6)	71 (17.8)
Constipation	5 (0.8)	4 (1.0)	37 (5.9)	42 (10.5)
Diarrhea	3 (0.5)	6 (1.5)	8 (1.3)	12 (3.0)^c^
Nausea	1 (0.2)	7 (1.8)	15 (2.4)	20 (5.0)
Vomiting	1 (0.2)	1 (0.3)	5 (0.8)	8 (2.0)
*General disorders and administration site conditions*	43 (7.1)	44 (11.3)	1 (0.2)	11 (2.8)
Injection site extravasation	12 (2.0)	12 (3.1)	0	0
Injection site pain	4 (0.7)	8 (2.1)	0	1 (0.3)
Injection site bruising	5 (0.8)	6 (1.5)	0	0
Injection site irritation	5 (0.8)	3 (0.8)	0	0
Injection site paresthesia	1 (0.2)	5 (1.3)	0	0
Injection site coldness	3 (0.5)	2 (0.5)	0	0
*Immune system disorders*	3 (0.5)	2 (0.5)	0	0
*Investigations*	20 (3.3)	5 (1.3)	10 (1.6)^c^	1 (0.3)
ALT increased	17 (2.8)	1 (0.3)	6 (1.0)	0
AST increased	14 (2.3)	0	8 (1.3)^c^	0
*Metabolism and nutrition disorders*	1 (0.2)	7 (1.8)	1 (0.2)	0
Hypophosphatemia	0	6 (1.5)	0	0
*Nervous system disorders*	21 (3.5)	28 (7.2)	12 (1.9)^c^	9 (2.3)
Dizziness	0	9 (2.3)	0	3 (0.8)^c^
Headache	12 (2.0)	13 (3.3)	9 (1.4)^c^	6 (1.5)^c^
Dysgeusia	4 (0.7)	3 (0.8)	0	0

AE = adverse event; ALT = alanine aminotransferase; AST = aspartate aminotransferase; FCM = ferric carboxymaltose; HMB = heavy menstrual bleeding; SMC = standard medical care.

^a^All comparisons between the FCM and SMC groups are statistically significant (*P* ≤ 0.05) unless otherwise noted.

^b^Each subject is counted only once per system organ class.

^c^Not statistically significant from the FCM group.

**Table 5:** Mean hemoglobin and ferritin levels at baseline and at the highest level after randomization (safety population).

	FCM		SMC	
	Postpartum (*n* = 606)	HMB (*n* = 390)	Postpartum (*n* = 623)	HMB (*n* = 399)
*Hemoglobin, mean (SD), g/dL*				
Baseline	10.20 (1.161)	9.34 (1.387)	10.11 (1.191)	9.40 (1.310)
Highest postrandomization result	12.53 (0.899)	11.68 (1.090)	11.97 (1.153)^a^	10.89 (1.246)^a^
*Mean ferritin (SD), ng/mL*				
Baseline	25.99 (22.730)	8.89 (15.529)	26.55 (24.973)	7.95 (15.072)
Highest postrandomization result	180.97 (96.798)	101.62 (89.312)	24.23 (16.743)^a^	20.94 (53.122)^a^

FCM = ferric carboxymaltose; HMB = heavy menstrual bleeding; SD = standard deviation; SMC = standard medical care.

^a^ *P* < 0.001 for between-group comparison.
